# Treatment with teriparatide for advanced bisphosphonate-related osteonecrosis of the jaw around dental implants: a case report

**DOI:** 10.1186/s40729-017-0074-6

**Published:** 2017-03-30

**Authors:** Yusuke Zushi, Kazuki Takaoka, Joji Tamaoka, Miho Ueta, Kazuma Noguchi, Hiromitsu Kishimoto

**Affiliations:** Department of Oral and Maxillofacial Surgery, Hyogo College of Medicine, 1-1 Mukogawa-cho, Nishinomiya, Hyogo 663-8501 Japan

**Keywords:** Bisphosphonate-related osteonecrosis of the jaw, Dental implant, Teriparatide

## Abstract

We report a case of a 66-year-old severely osteoporotic woman with bisphosphonate-related osteonecrosis of the jaw (BRONJ) around her dental implants, who was treated successfully with teriparatide and sequestrectomy of the mandible. After 5 months of teriparatide therapy, the sequestrum separation had progressed and a sequestrectomy was performed under general anesthesia. Five months after the operation, new bone formation was observed around the bone defect in the region of the sequestrectomy. A repeat computed tomographic image revealed improvement in the bone defect in the mandible. These results suggest that teriparatide provides beneficial effects in the treatment of advanced BRONJ around dental implants.

## Background

Oral bisphosphonates (BPs) are used to treat osteoporosis, Paget’s disease, and osteogenesis imperfecta. They are most widely used for treatment of osteoporosis. BP-related osteonecrosis of the jaw (BRONJ) was first reported by Marx in 2003 [1]. The risk of BRONJ in osteoporotic patients treated with BPs remains low compared with that of oncology patients [2]. Recent studies have indicated that the relative incidence of BRONJ in patients with osteoporosis is higher than previously thought [3]. Madrid and Sanz [4] suggested that the placement of dental implants in patients treated with oral bisphosphonates was not associated with the onset of BRONJ; they found no relationship between the treatment and the survival of implants. However, more recently, an increasing number of peri-implant BRONJ cases have been described [5–9]. Peri-implant BRONJ currently is considered an additional complication related to oral implants, along with nerve injury, bleeding, sinus perforation, implant ingestion/aspiration, peri-implantitis, and mucositis [6].

We present a case of a severely osteoporotic woman with BRONJ around her dental implants, who was treated successfully with teriparatide and sequestrectomy.

## Case presentation

A 66-year-old woman was referred to the Oral and Maxillofacial Surgery Clinic at Hyogo College of Medicine Hospital, Japan, in September 2011, for an extraoral fistula and refractory pain of the right mandible associated with a purulent discharge and soft tissue swelling. The patient’s osteoporosis was diagnosed in 2005 and treated with 35 mg of alendronate weekly by the family doctor. The patient had a past history of severe osteoporosis, multiple vertebral fractures, and renal failure. She had taken 20 mg of prednisone for 3 months from 2005 for the treatment of IgA nephropathy.

Dental implant treatment in the maxilla and mandible was begun in June 2009 by the family dentist. Five implants (Spline Twist implant, Zimmer Dental, Carlsbad, CA) were placed at the same time in the posterior region of the mandible. The surgical procedure was uneventful, and primary stability of the implants was achieved. In September 2010, at the time of implant reopening for the second surgery, the implants had integrated and the healing abutments were connected. Provisional maxillary and left mandibular prostheses were cemented onto the abutments.

Nine months after the second surgery in June 2011, the patient started to complain of a painful cheek swelling on the right side of the mandible, associated with gingival bleeding. She was prescribed oral antibiotics by her dentist and underwent occasional antibiotic therapy thereafter.

**Fig. 1:**
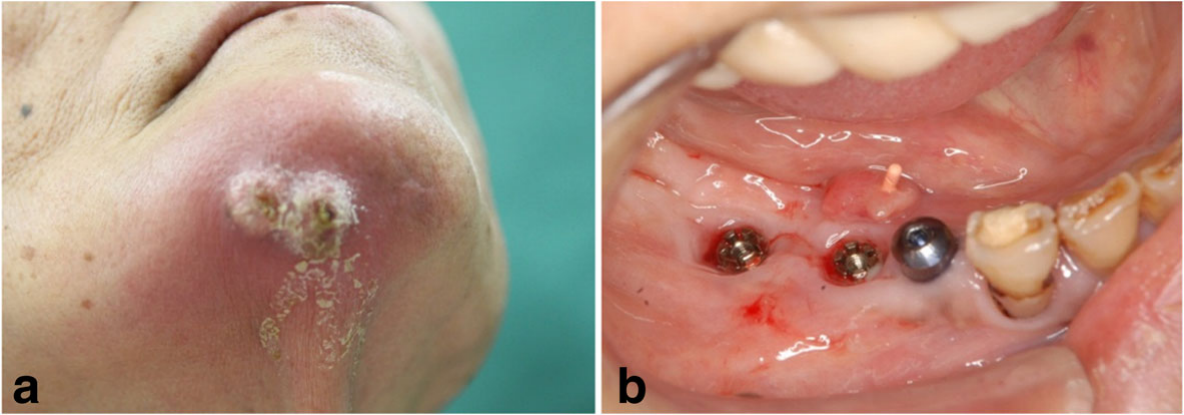
**a** Extraoral photograph showing an extraoral fistula in the right mandibular region. **b** Intraoral photograph showing an intraoral fistula on the lingual side of the distal dental implants associated with mucosal inflammation and a purulent discharge

**Fig. 2:**
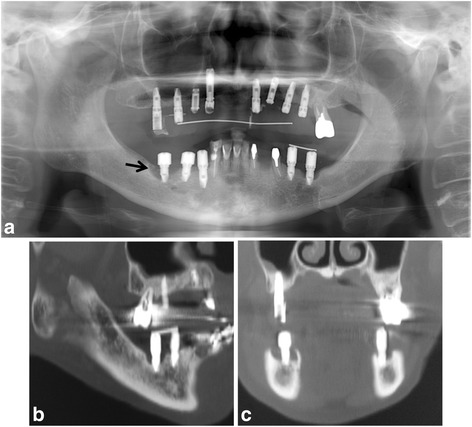
**a** Panoramic radiograph showing marked alveolar bone resorption surrounding the dental implant replacing the right mandibular first molar (*arrow*). **b** Sagittal CT view. **c** Coronal CT view

In September 2011, the patient was referred to our clinic because her symptoms were getting worse. Clinical examination revealed an intraoral fistula on the lingual side of the dental implant replacing the right mandibular first molar, associated with mucosal inflammation and a purulent discharge (Fig. 1a, b). She also had hypoesthesia of the right lower lip. The patient underwent panoramic radiography (Fig. 2a) and computed tomography (CT), which showed bone resorption around the dental implant in the right mandibular first molar area and severe peri-implantitis in the right mandibular molar region. There was no obvious sequestrum separation (Fig. 2b, c). Under a clinical diagnosis of perimandibular inflammation and peri-implantitis, conservative treatment consisting of local irrigation and use of antibiotics was implemented. Meropenem hydrate was given initially, then changed to ampicillin/sulbactam. The inflammatory state improved, and when the symptoms subsided, treatment with clarithromycin was continued. Debridement and removal of the dental implant in the right mandibular first molar area was performed under local anesthesia. Irrigation of the site was continued as part of the treatment regimen.

In November 2011, after a consultation with an osteoporosis expert at the Orthopedic Medicine Clinic of our hospital, alendronate therapy was stopped and subcutaneous teriparatide therapy at a dose of 20 μg per day was started. During the course of the teriparatide therapy, the patient continued to use 0.02% benzalkonium chloride solution for local irrigation.

**Fig. 3:**
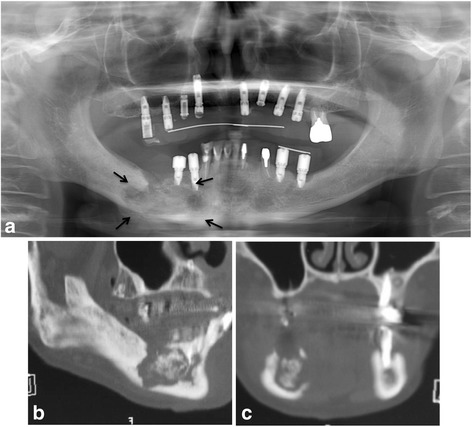
**a** Panoramic radiograph showing the sequestrum separation after 5 months of teriparatide therapy (*arrows*). **b** Sagittal CT view. **c** Coronal CT view

**Fig. 4:**
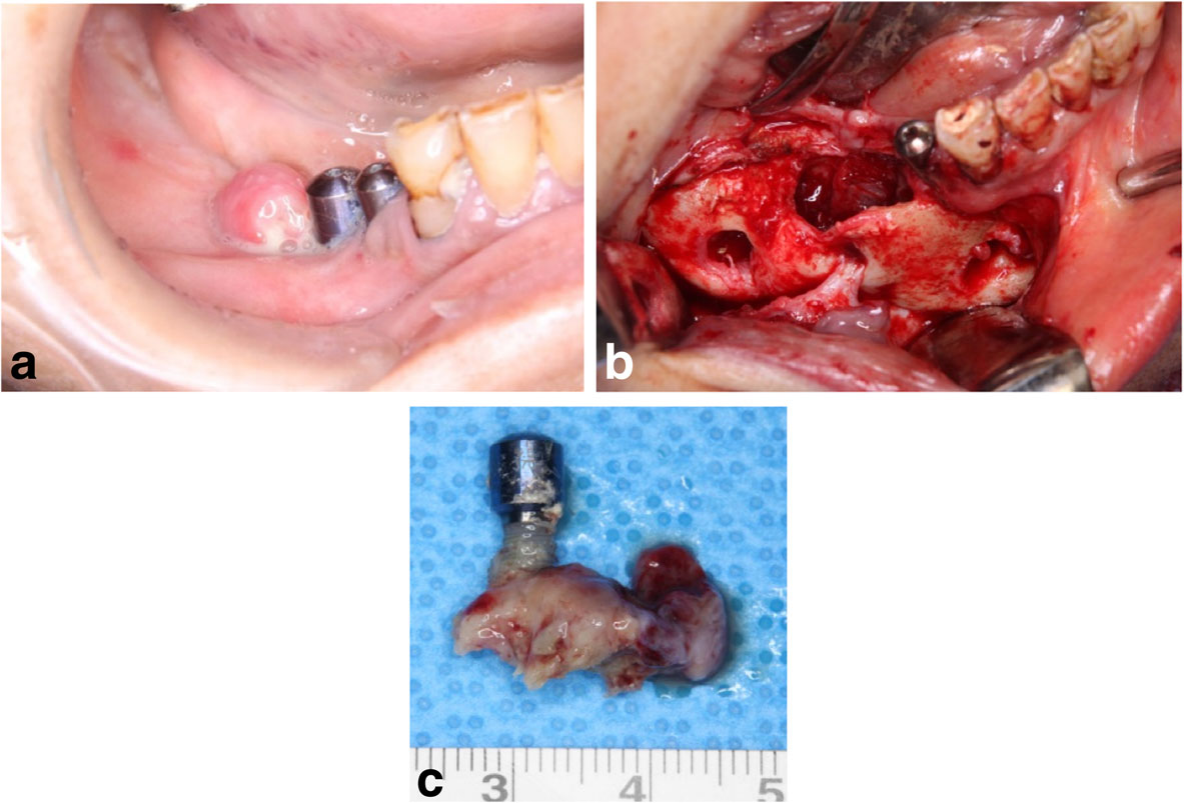
**a** Preoperative intraoral photograph. **b** Intraoperative photograph of the sequestrectomy. **c** Removal of the dental implant with a specimen of the necrotic bone

**Fig. 5:**
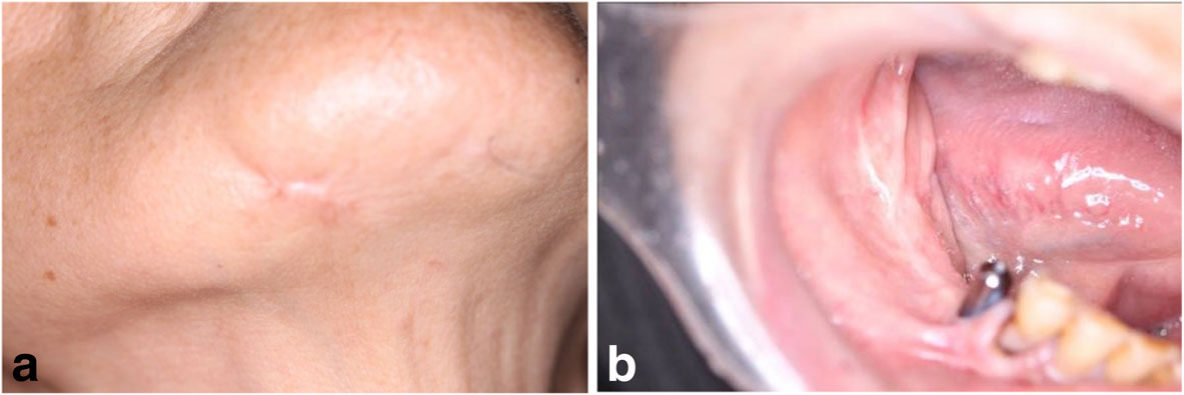
**a** Extraoral photograph 5 months after the sequestrectomy. **b** Intraoral photograph 5 months after the sequestrectomy.

**Fig. 6:**
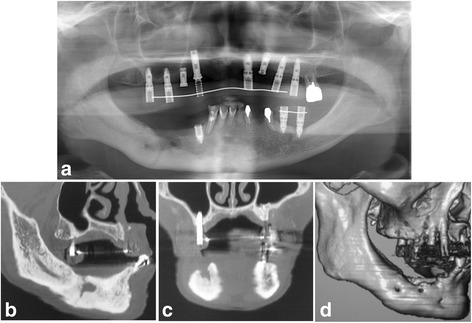
**a** Panoramic radiograph 16 months after the sequestrectomy. **b** Sagittal CT view. **c** Coronal CT view. **d** 3D CT view

In April 2012, after 5 months of teriparatide therapy, the sequestrum separation had progressed (Fig. 3), and a sequestrectomy was performed under general anesthesia (Fig. 4). At 5 months after the operation, a CT scan revealed new bone formation around the bone defect in the region of the sequestrectomy, with all symptoms including bone exposure disappearing (Fig. 5). The patient’s osteoporosis treatment was continued, and 16 months after the sequestrectomy, further new bone formation was observed (Fig. 6).

## Discussion

We describe a case of a patient with a 6-year history of alendronate therapy, in which BRONJ developed around her dental implants. In this patient, the dental implants achieved successful osseointegration, and BRONJ occurred after the second surgery. Several factors could have played a role in the development of BRONJ in this patient. Glucocorticoid therapy is associated with an increased risk of BRONJ. This may be a result of multiple factors including inhibition of osteoblast function and increased osteoblast and osteocyte apoptosis. Other effects of glucocorticoids that may contribute to an increased risk of BRONJ include increased bone resorption, immunosuppression, impaired wound healing, and increased risk of local infection [10]. Patient-related local risk factors include dentoalveolar surgery (e.g., tooth extraction) and pre-existing inflammatory dental disease, such as periodontal disease or periapical pathology [11]. Although BPs tend to accumulate in sites of active bone remodeling, such as the jaws, the surgical trauma to the alveolar bone during implant surgery could have further stimulated the postoperative accumulation of the drug in the implant site. The localized interference of BPs on bone turnover may have influenced the peri-implant bone resistance to oral bacteria in the long term, thus increasing the risk of peri-implantitis. Once infection of the implanted bone site is established, BPs further accumulate because of the increased bone turnover; the onsite activation of bisphosphonates will hamper the healing capacity of bone, leading to bone necrosis and sequestration [5].

Nevertheless, the role of the dental implant procedure as a BRONJ pathogenetic factor [12–15] is still unclear. Recently, an increasing number of peri-implant BRONJs have been described [5–9]. Peri-implant BRONJ has been classified into two types: implant surgery-triggered BRONJ, when it develops within 6 months after implant surgery, suggesting that the surgical process may be a contributing factor; and non-implant surgery-triggered BRONJ, if it develops 6 months or more after implant surgery, or when BP administration started after implant placement and osteointegration [8]. Most authors do not consider the surgical procedure of implantation as a trigger factor for MRONJ [7, 8, 14, 16–20].

It is therefore important that all patients treated with oral BPs must be given a full explanation of the potential risks of implant failure and BRONJ development in the short and long term. Because the potential role of infection in implant failure and BRONJ occurrence is still debated, great attention should be paid to the long-term oral hygiene and plaque control of implant-prosthetic restorations in patients taking oral BPs.

BPs and other antiresorptives such as denosumab increase apoptosis and inhibit osteoclast differentiation and function, all leading to decreased bone resorption and remodeling [11]. Teriparatide may counteract these mechanisms by stimulating bone remodeling. It has been shown to stimulate the activity and viability of osteoblasts from the alveolar bone of chronic bisphosphonate users [21], while indirectly increasing the metabolic activity and number of osteoclasts by affecting osteoblast function [22]. An increased number of remodeling units and increased bone formation within each unit may promote healing and the removal of damaged bone. Thus, teriparatide may offer therapeutic promise for localized bone defects of the jaw in patients with BRONJ [3, 23–25].

While it has been suggested recently that assertive surgical removal of the sequestrum appears to be effective [26–29], it can sometimes be difficult to distinguish living bone from necrotic bone. Recently, resection of BRONJ-affected tissue produced healing in patients taking oral bisphosphonates more successfully than conservative management [30]. However, bone resection because of surgical treatment may lead to significant oral disability.

Activation of living bone turnover by teriparatide therapy causes progression of the separation of the sequestrum. As a result, teriparatide therapy promotes sequestrum separation followed by normal mucosal coverage of the exposed bone. After 5 months of teriparatide therapy in our patient, sequestrum separation had progressed and thus a sequestrectomy was performed under general anesthesia. After the wound in the affected area had healed, our patient did not report any problems pertaining to her ability to ingest food, despite the presence of the bone defect in the mandible. We treated the patient with teriparatide for 2 years. CT monitoring of the mandible would assist in determining whether teriparatide can allow complete recovery of the bone defect in the mandible in cases of ONJ induced by bisphosphonates.

## Conclusions

We have reported a case of a severely osteoporotic elderly woman with BRONJ around her dental implants, who was treated successfully with teriparatide. Teriparatide therapy appeared to exert beneficial effects in this patient.
